# Calcium release and physical properties of modified carbonate apatite cement as pulp capping agent in dental application

**DOI:** 10.1186/s40824-018-0146-6

**Published:** 2018-12-06

**Authors:** Myrna Nurlatifah Zakaria, Arief Cahyanto, Ahmed El-Ghannam

**Affiliations:** 1 Department of Endodontology and Operative Dentistry, Program Study of Dentistry, Faculty of Medicine, Universitas Jenderal Achmad Yani, Cimahi, Indonesia; 2 Department of Dental Materials Science and Technology, Faculty of Dentistry, Universitas Padjadjaran, Sumedang-Jatinangor, Indonesia; 3 Department of Mechanical Engineering and Engineering Science, The University of North Carolina at Charlotte, Charlotte, NC USA

**Keywords:** Carbonate apatite, Silica-calcium phosphate composite, CO_3_Ap-SCPC cement, Pulp capping

## Abstract

**Background:**

Carbonate apatite (CO_3_Ap) and silica-calcium phosphate composite (SCPC) are bone substitutes with good prospect for dental application. SCPC creates a hydroxyapatite surface layer and stimulate bone cell function while, CO_3_Ap induce apatite crystal formation with good adaptation providing good seal between cement and the bone. Together, these materials will add favorable properties as a pulp capping material to stimulate mineral barrier and maintain pulp vitality. The aim of this study is to investigate modification of CO_3_Ap cement combined with SCPC, later term as CO_3_Ap-SCPC cement (CAS) in means of its chemical (Calcium release) and physical properties (setting time, DTS and pH value).

**Methods:**

The study consist of three groups; group 1 (100% calcium hydroxide, group 2 CO_3_Ap (60% DCPA: 40% vaterite, and group 3 CAS (60% DCPA: 20% vaterite: 20% SCPC. Distilled water was employed as a solution for group 1, and 0.2 mol/L Na_3_PO_4_ used for group 2 and group 3.

Samples were evaluated with respect to important properties for pulp capping application such as pH, setting time, mechanical strength and calcium release evaluation.

**Results:**

The fastest setting time was in CO_3_Ap cement group without SCPC, while the addition of 20% SCPC slightly increase the pH value but did not improved the cement mechanical strength, however, the mechanical strength of both CO_3_Ap groups were significantly higher than calcium hydroxide. All three groups released calcium ions and had alkaline pH. Highest pH level, as well as calcium released level, was in the control group.

**Conclusion:**

The CAS cement had good mechanical and acceptable chemical properties for pulp capping application compared to calcium hydroxide as a gold standard. However, improvements and in vivo studies are to be carried out with the further development of this material.

## Background

Silica-calcium phosphate composite (SCPC) and carbonate apatite (CO_3_Ap) are bioceramics material that has been intensively studied for bone repair [1–8]. CO_3_Ap is a full transformed cement with high solubility to be replaced by new bone formation, oscteoclastic bone resorption followed by new bone formation was observed in implanted CO_3_Ap granules in bone defect [1, 9]. Fabrication of carbonate apatite cement from vaterite and dicalcium phosphate anhydrous (DCPA; CaHPO_4_) powder mixed with various sodium phosphate solutions: NaH_2_PO_4_, Na_2_HPO_4_, and Na_3_PO_4_ showed that transformation rate of cement to form CO_3_Ap was affected by the solutions pH. Therefore, 0.2 mol/L Na_3_PO_4_ solution with pH 12.3for fastest CO_3_Ap transformation rate compared to the others was used in this present study [2]. One of the advantages of CO_3_Ap cement is that it can set in the physiological condition through a dissolution-precipitation reaction. After mixing with Na_3_PO_4_ solution, the vaterite and DCPA dissolved to supply Ca^2+^, PO_4_^3−^, and CO_32−_ ions, which play important role in mineralized tissue regeneration as well as in dentinogenesis [10, 11].

SCPC withdrew Ca^2+^ ions from it surrounding to its surface which provides Ca^2+^ ions for osteoblast activity, by facilitating osteoblast to differentiate to mature bone-forming cells [7, 12]. SCPC has been implanted to alveolar bone socket after tooth extraction and after 6 months a bone core was taken for histological evaluation followed by an implant placement. Histomorphometric analysis showed good regeneration of new vital bone and radiographic assessment immediately after extraction compared to 6 months after extraction and grafted by SCPC granules showed a minimum changed in alveolar ridge dimensions [6]. An in vitro study demonstrated that SCPC exhibited a controlled release of silicon and phosphate ions in cell culture media delivering a natural stimulus for bone-cell differentiation [13, 14].

The pulp is a loose connective tissue in the central of a tooth, with various cells including fibroblast, odontoblast, immune cells, undifferentiated mesenchymal cells, sensory nerves and blood vessels [15]. It plays a pivotal part in maintaining the vitality of the tooth for regenerative, sensory, and nutritive function. If an injury to the enamel or dentin occurs, the pulp will protect itself by pumping immune cells to injured area, as well as stimulating the odontoblast to form a reparative or reactive dentin in order to block the irritants, preventing harm to reach to the pulp and maintain a vital and healthy pulp with no irreversible pulp inflammation [15–18]. However, the pulp can be injured due to enamel and dentin fracture, or progressive dental caries resulting pulp exposure, causing irreversible inflammation and eventually causing pulp necrosis. This will act as a port of entry for bacteria to colonize the pulp space inducing periapical lesion (periapical abscess, cyst, granuloma), therefore in these kind of situation, root canal treatment will be the only treatment choice besides tooth extraction [16, 17].

Treatment choice for exposed dental pulp to save its vitality is called pulp capping. Pulp capping is a treatment by placing a biocompatible material on the exposed pulp to stimulate the formation of mineralized dentinal bridge or reparative dentin prior the definite restoration. The gold standard material for this purpose is calcium hydroxide [Ca(OH)_2_] which has been used from the early 1920s, for its biocompatibility and ability to stimulate hard tissue formation as well as antibacterial effect [17]. The liberation of hydroxyl and calcium ions is believed to be the basic mechanism for its high pH contributing to antibacterial effect and activation of alkaline phosphates (ALP) involved in hard tissue formation [16–20]. Some drawbacks concerning this compound including its high solubility, degradation of material over time, weak dentinal barrier formation, poor adhesion property to tooth structure, and long uncontrolled chronic inflammation to the pulp [19, 21–24].

The tooth and bone, are principally similar in their structure, in which they are mainly composed of hydroxyapatite [Ca_10_(PO_4_)_6_(OH)_2_] crystal, carbonate-substituted as the major part of their inorganic constituent. Pondering the same major component of dentin and bone, we proposed the used of CO_3_Ap and SCPC as novel pulp capping materials that are biocompatible, bioactive and able to set in the physiological environment to induced mineralization over the expose pulp and maintain the pulp vitality. Our previous study has already evaluated the different ratio of CO_3_Ap and SCPC for the best formula for this purpose [22]. The present study is a sequel of previous one, to evaluate the physical properties (pH, setting time, DTS) and Ca^2+^ ions release of the new CO_3_Ap-SCPC cement as new pulp capping material.

## Methods

### Preparation of CO_3_Ap and SCPC powders

The CO_3_Ap powder composed in this study consists of DCPA (J.T. Baker Chemical Co., NJ, USA) and vaterite. The DCPA powder was grinded to reduce the particle size to 0.4 μm using a planetary ball mill (Fritsch 8 6560, Idar-Oberstein, Germany) with 95% ethanol for 1 h and drying for 3 h. The vaterite powder was prepared according to the previous report [25]. In brief, 50 g of Ca(OH)_2_ was put into 500 mL of methanol and 25 mL of distilled water. The CO_2_ gas was blown at a rate of 1 L/min for 120 min into the suspension and the temperature was set at 20 °C. The obtained particles were collected and dried at 110 °C. The average particle size of vaterite powder was approximately 0.7 μm. The SCPC used to modified the CO_3_Ap consists of 19.49% SiO_2_, 20.34% P_2_O_5_, 40.68% CaO and 19.49% Na_2_O (in mol %) as it demonstrated favorable physiochemical and bioactivity properties of Si-rich SCPC [13]. The powder metallurgy technique was employed to prepare the SCPC powders. The powders were mixed in polyethylene bottles over a roller for 24 h, then calcinated at 800 °C (Thermolyne 30,400, Barnstead International, Dubuque, IA) for 1 h, and then ground to the size average of 90–150 μm.

### Preparation of Ca(OH)_2_ powder

In this study, a commercial Ca(OH)_2_ powder (Merck, Darmstadt, Germany) was employed as a control. Sample manipulation was done as the manufacturer instruction.

### Preparation of the samples

The powder cement ratio divided into 3 groups: group 1 (100% Ca(OH)_2_) as a control, group 2 (60% DCPA: 40% vaterite), and group 3 (60% DCPA: 20% vaterite: 20% SCPC). Distilled water was used as solution for group 1, and 0.2 mol/L Na_3_PO_4_ used for group 2 and group 3.

### Setting time

All group samples were prepared at liquid-to-powder (L/P) ratios of 0.5. Setting time of all samples were measured according to the method set in ISO 1566 for dental zinc phosphate cement. In this method, a cement is considered to set when a 400 g weight loaded onto a Vicat needle with a tip diameter of 1.0 mm fails to make a perceptible circular indentation on the surface of the cement. The standard requires the cement to be maintained at a temperature of 37 °C and 100% relative humidity to simulate the clinical condition. In the present study, the specimen was placed on Teflon mold for setting time measurement. Mean setting time (*n* = 5) was obtained, and the standard deviation was used as an evaluation of standard uncertainty.

### pH measurement

pH was observed using pH meter (pH -207, Lutron Co., Taipei, Taiwan). Before measurement, pH meter was calibrated by a buffer solution of pH 4 and 7. After calibrated, pH solution was measured by putting the electrode inside the tube with a sample and deionized water for 0.5, 1, 24, 72 and 168 h.

### Calcium released

The calcium released was examined using spectrophotometer UV/VIS (Spectronic Camspec Ltd., Leeds, UK). The set cement sample was put into a tube filled with 10 mL of deionized water at 37 °C. The stored water was measured for Ca^2+^ ions analysis for 0.5, 1, 24, 72 and 168 h.

The formula to measure calcium released was below:

$$ Calcium\ \left(\frac{mmol}{L}\right)=\frac{\mathrm{Abs}.\mathrm{Test}}{\mathrm{Abs}.\mathrm{Std}}x\  Standard $$

### Mechanical strength measurement

The mechanical strength of the samples was examined in terms of diametral tensile strength (DTS). Each group was mixed with the different aqueous solution, at liquid to powder ratio of 0.5 and set at 37 °C and 100% of relative humidity for 72 h. The paste was put into a Teflon mold (6 mm in diameter × 3 mm in height), both ends of the mold were covered with glass slides then clamped. The molds were placed inside a plastic container with distilled water to maintain 100% relative humidity. The plastic container was placed into an incubator and kept at 37 °C for 72 h. The samples were removed from the mold after completion of treatment times and immersed in the 99% ethanol for 3 min then dried at 80 °C for 3 h. Then, the samples were crushed using a universal testing machine (LRX Plus; Llyod Instruments, Ltd., West Sussex, UK) with 5.6 kN of preload stress at a crosshead speed of 1 mm/min. DTS values were taken as the average of at least 5 samples.

## Results

Table 1 summarizes the setting time comparison between Ca(OH)_2_, CO_3_Ap and CAS. The Ca(OH)_2_ was mixed with distilled water as a control, CO_3_Ap and CAS was mixed with 0.2 mol/L Na_3_PO_4_, respectively. The setting time of each group were statistically different where the CO_3_Ap group had the fastest setting time (13.08 ± 0.05 min) followed by CAS and Ca(OH)_2._

**Table 1:** Comparison of calcium hydroxide, carbonate apatite and carbonate apatite-SCPC on setting time evaluation (*n* = 5)

Liquid	Powder	Setting time (minutes)
		L/P 0.5
Distilled water	Calcium hydroxide	38.05 ± 0.12
Na_3_PO_4_	Carbonate apatite^*^	13.08 ± 0.05
	Carbonate apatite-SCPC^**^	26.93 ± 0.21

*n = 5;* ^*/**^ *p < 0.05*

**Fig. 1:**
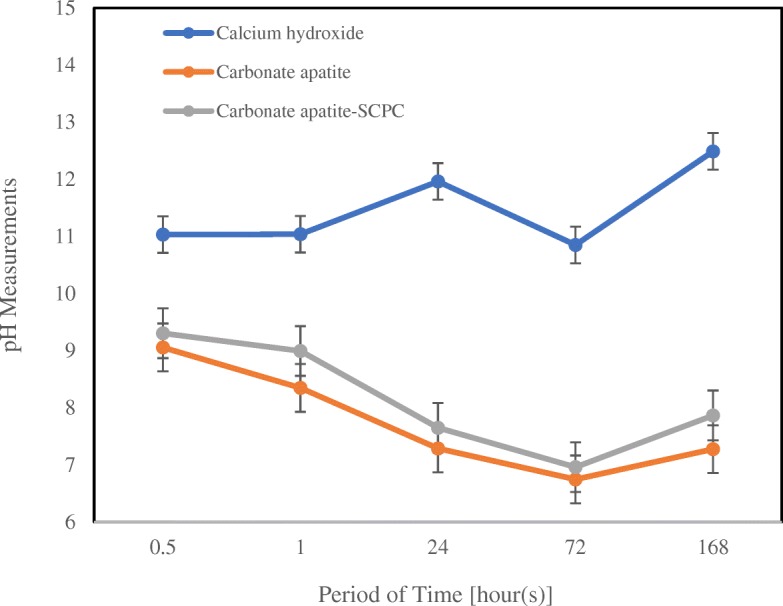
pH values for three different materials in various period of time. At least 5 samples were measured for pH. Errors bars indicate the standard deviation

The mean pH value and standard deviations recorded for three different material group at the various period of time are plotted in Fig. 1. In this study, calibration of pH was conducted using buffer solution before pH measurement and were similar as buffer control. The Ca(OH)_2_ revealed higher pH values than other samples at all period of time evaluation (*p < 0.05*). The pH values of CO_3_Ap and CAS were similar to each other and the pH values in these both groups gradually decreased until near neutral at 168 h.

**Fig. 2:**
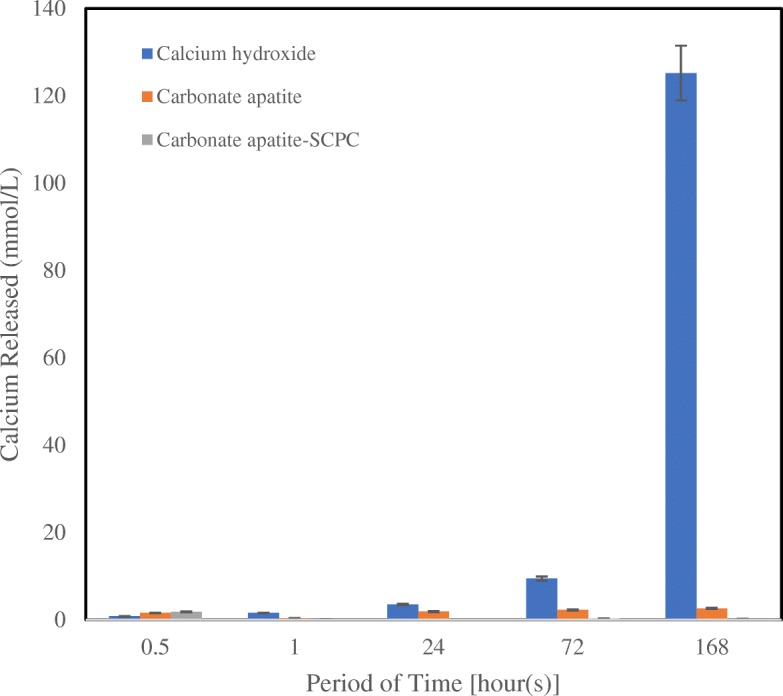
The concentration of Calcium released from the set of three different materials. At least 5 samples were measured for calcium released. Errors bars indicate the standard deviation

Figure 2 presents the mean Ca^2+^ ions released and standard deviations provided by three different materials as a function of time. The Ca(OH)_2_ released 0.81 mmol/L Ca^2+^ ions in the first 0.5 h and increased significantly to 125.20 mmol/L at 168 h. The Ca^2+^ ions released for CO_3_Ap group showed stable results in all the period of time. While, CAS had higher Ca^2+^ ions released in the first 0.5 h (1.84 mmol/L). However, the Ca^2+^ ions released on CAS group gradually decreased until 0.01 mmol/L at 24 h.

**Fig. 3:**
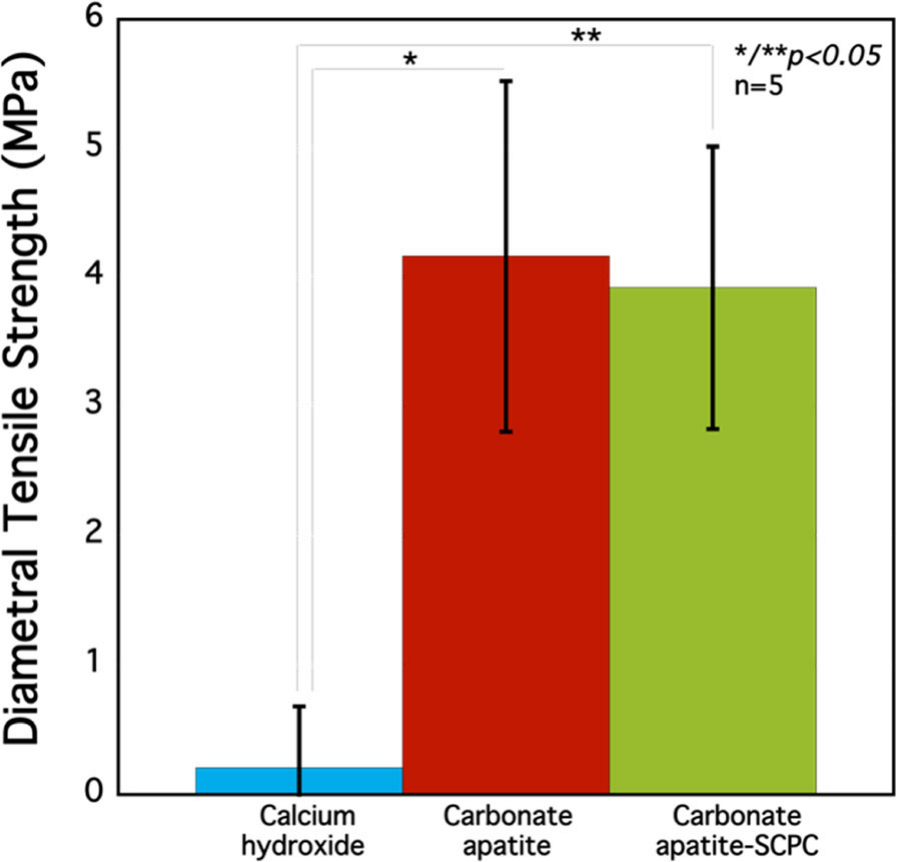
Diametral tensile strength values of the set three different materials after 72 h. At least 5 samples were measured for DTS evaluation. Errors bars indicate the standard deviation

Figure 3 shows the mean of DTS values of three different materials after setting at 37 °C, 100% relative humidity for 72 h. The mean DTS values of CO_3_Ap (4.16 ± 1.36 MPa) and CAS (3.92 ± 1.07 MPa) were statistically higher than Ca(OH)_2_ (0.21 ± 0.47 MPa). However, there was no statistical significance on DTS values between CO_3_Ap and CAS.

## Discussion

The setting of the CO_3_Ap cement lies on dissolution-precipitation reaction. After mixing with 0.2 mol/L Na_3_PO_4_ solution, the powder will dissolve and supply Ca^2+^, PO_4_^3−^ and CO_3_^2−^ ions. As the solution gets supersaturated, precipitation occurs and forms CO_3_Ap crystals and the cement will set [1, 9, 10]. As shown in Table 1 the setting time for CO_3_Ap cement without the addition of SCPC is significantly higher than CO_3_Ap and Ca(OH)_2_ cement. The CO_3_Ap cement consists of higher percentage of vaterite that has high solubility thus enabling precipitation of CO_3_Ap in a shorter time. This was also one of the reasons for employing vaterite instead of calcite in this study. On the other hand, SCPC added in group 3 were in the form of granules that do not dissolve in short time, they do release Ca, P, Na, and Si ions but they do not have the ability to set.^8^ This was also the reason why we combine the SCPC with CO_3_Ap instead of using pure SCPC for pulp capping purpose.

To achieve a favourable cement to be applied as pulp capping agent, adequate setting time and the ability to set in a moist environment with the presence of blood or blood clot will give the best result, because the material will hold place without being washed out. This is one of Ca(OH)_2_ drawback, because it is easy to get dissolved and have a poor adhesive ability to tooth structure. An ideal pulp capping material should have the ability to adapt and adhere to the tooth structure, providing tight seal to prevent microleakage and bacterial penetration [17, 26].

The longest setting time for Ca(OH)_2_ relates on the high solubility and could also relate to its larger particle size (37 μm), compared to DCPA (0.3–0.5 μm) and vaterite (0.6–0.9 μm). The Ca(OH)_2_ setting mechanism is based on acid–base reaction between Ca(OH)_2_ and 1-methyl trimethylene disalicylate, forming an amorphous calcium-disalicylate salt, with Ca^2+^ ions intercalated with disalicylate molecules. For faster setting time, commercially Ca(OH)_2_ products added accelerator in the form base and catalyst paste, enabling more predictable and faster setting time, however, this approach did not solve the adhesive problem. Meanwhile, the addition of SCPC to CO_3_Ap cement slightly decreased the mechanical strength with no significant difference. In line with our previous study, the addition of 10 and 20% of SCPC did not gave significant difference and had better mechanical strength compared to 10 and 40% addition of SCPC. The bioactive silicate functional groups of SCPC appeared to bind the CO_3_Ap components in the CAS cement and formed a more compact and dense structure as seen in the SEM analyses [22].

The pH evaluation showed a trend of pH value that slowly decreases over time in both CO_3_Ap cement group with or without SCPC, whereas Ca(OH)_2_ have a relatively stable pH level until 168 h observation. This showed that, after precipitation and the formation of the CO_3_Ap crystal, pH decreases, though did slightly elevate at 168 h evaluation and tend to be at neutral level. However, the addition of SCPC to CO_3_Ap cement did not gave any significant effect on the pH level. This is completely different compared to the Ca(OH)_2_ group that still have relatively high pH level (above 11) for all evaluation time. The relatively high pH for Ca(OH)_2_ group are closely related to the dissolution of Ca^2+^ and OH^−^ ions after the powder was manipulated with distilled water. Alkaline pH of the dental material is strongly associated with its antimicrobial effect because the alkaline environment will interfere bacterial proliferation [17, 26–28]. Alkaline pH also contribute to the increase expression of bone morphogenetic protein-2 (BMP-2), alkaline phosphatase (ALP), and promotes the formation of calcified nodules favoring the tissue environment to stimulate the healing process [18, 25, 27].

Ca^2+^ ions also play an important role in the formation of the dentinal bridge. Ca^2+^ ions stimulate the recruitment and proliferation of undifferentiated cells from the pulp and activate stem cells and odontoblast to form reparative dentin or dentinal bridge to protect an exposed pulp [27, 29]. Moreover, it enhances pyrophosphatase activity, sustaining dentin mineralization and dentinal bridge formation [30]. The bioactivity of a material is affected by the nature structure and surface of the material responsible for water sorption, material solubility and its permeability water diffusion (i.e., porosity) [27]. As expected, the significant difference was observed between the CO_3_Ap and CAS cement to Ca(OH)_2_ cement that rapidly dissolves. The high solubility of Ca(OH)_2_ make less effective for application in the presence of blood which typically occurs in exposed pulp, especially in heavily inflamed ones. In general, slow release bioactivity is beneficial to have a balance between material resorption to regeneration, this would probably be hard to achieve in Ca(OH)_2_ and could be the reason for its weak and tunnel defects on their dentinal bridge formation. However, over stabilization can also result in pointless ionic dissolution.

## Conclusion

Based on our findings of acceptable setting time, alkaline pH and calcium release, together with previous study on its ability to form reparative dentin in animal study, the novel CAS cement can be considered as a good candidate for pulp capping treatment. Nevertheless, further evaluation on improving the properties still awaits.
